# Restoring walking ability in older adults with arm-in-arm gait training: study protocol for the AAGaTT randomized controlled trial

**DOI:** 10.1186/s12877-023-04255-9

**Published:** 2023-09-06

**Authors:** Mathilde Gigonzac, Philippe Terrier

**Affiliations:** Haute Ecole Arc Santé, HES-SO University of Applied Sciences and Arts Western Switzerland, Neuchâtel, Switzerland

**Keywords:** Human locomotion, Aging, Falls, Physical activity, Walking, Gait training

## Abstract

**Context:**

Falls are a significant problem among older adults. While balance and functional exercises have been shown to be effective, it remains unclear whether regular walking has specific effects on reducing the risk of falls.

**Rationale:**

Older people who fall frequently have impaired gait patterns. Recent studies have suggested using interpersonal synchronization: while walking arm-in-arm, an older person synchronizes steps with a younger person to reinstate a better gait pattern. This method of gait training may reduce the risk of falls.

**Objective:**

The aim is to assess the efficacy of an arm-in-arm gait-training program in older people.

**Design:**

The arm-in-arm gait training trial (AAGaTT) is a single-site, open label, two-arm, randomized controlled trial.

**Participants:**

We will enroll 66 dyads of older people and their younger “gait instructors”. The older participants must be > 70 years old with adequate walking ability. They must have experienced a fall in the year prior to study entry.

**Intervention:**

Dyads will walk an indoor course for 30 min either side-by-side without contact (control group) or arm-in-arm while synchronizing their gait (intervention group). The gait training will be repeated three times a week for four weeks.

**Outcomes:**

The main outcome will be the walking speed measured in five-minute walking trials performed at baseline and at the end of each intervention week (week 1 – week 4), and at week 7. Gait quality will be assessed using accelerometers. We will also assess perceived physical activity and health using questionnaires. Finally, we will monitor fall incidence over 18 months. We will evaluate whether outcomes are more improved in the intervention group compared to the control group. In addition, interviews will be conducted to assess the perception of the gait training.

**Expected results:**

Recent advances in the neurophysiology of motor control have shown that synchronizing gait to external cues or to a human partner can increase the efficiency of gait training. The expected benefits of arm-in-arm gait training are: reduced risk of falls, safe treatment with no adverse effects, and high adherence. This gait training program could be a low-cost intervention with positive effects on the health and well-being of seniors.

**Trial registration:**

ClinicalTrials.gov NCT05627453. Date of registration: 11.25.2022.

## Background

In the context of an aging population, the high incidence of falls among older people is a cause for concern. Approximately one in three older people falls each year [1]. In addition to high direct costs, the consequences of falls include functional deficits, loss of mobility, and impaired quality of life [2]. Strategies can be implemented to reduce the incidence of falls and their negative consequences if those at risk are identified early [3–5]. A recent systematic review concluded that exercise programs reduce the rate of falls and the number of older people who fall (high certainty evidence) [5]. Effective interventions usually consist of balance and functional exercises. It is still unclear whether regular walking has specific effects on reducing the risk of falling [5, 6]. It is not excluded that incorporating more walking into the daily lives of older people may expose them to more opportunities to fall [5]. Making walking safer could be one solution.

Walking while synchronizing gait to rhythmic auditory cues, such as walking to the beat of upbeat music, has substantial benefits for improving walking capacity in older adults. Initially proposed to treat specific pathologies such as Parkinson's disease [7], rhythmic gait training also benefits the general older population in terms of gait performance (faster speed and longer strides) [7]. Similarly, a meta-analysis showed that rhythmic gait training may increase walking speed [8]. In patients with Parkinson’s disease, recent evidence from a randomized controlled trial suggests that rhythmic gait training may reduce the number of falls [9]. However, it remains uncertain whether this beneficial effect on fall risk generalizes to all older people. It has also been proposed to improve locomotor function by synchronizing gait with other types of sensory cues [10]. For example, aligning steps to visual cues displayed on the walking surface is an effective means of improving gait in Parkinson’s disease [11, 12]. Training with visual feedbacks is also used to improve static and dynamic balance, with likely benefits in the older population [13]. Overall, there is strong evidence to support the use of external cues, either auditory or visual, to improve the efficacy of gait training in older people. However, further studies are needed to better define the most efficient way to deliver such gait training and to confirm its true effectiveness in reducing falls.

The steady-state human gait pattern exhibits a small amount of variability between successive strides. For example, the duration and length of consecutive strides vary by a few percent over a half hour of walking at the preferred speed [14, 15]. These fluctuations are not random, but follow a correlated pattern; that is, a stride longer than the average is more likely to be followed by another longer stride [14]. Furthermore, these correlations extend over dozens of consecutive strides and decrease according to a power law (1/f noise, fractal pattern [16]). It has been suggested that external cues that mimic this pattern may be more effective than classical (isochronous) cues. Recent studies have shown that walking to a “fractal metronome” is effective in inducing a more natural stride time structure than walking to an isochronous metronome in patients with Parkinson's disease [17–19].

Because synchronizing gait with rhythmic cues has beneficial effects, it has been proposed to use interpersonal synchronization to restore locomotor function [20]. An individual with gait deficits could walk side-by-side with a healthy individual. The unhealthy individual would synchronize his or her gait with that of the healthy partner and could thus benefit from these "human cues" in a manner similar to that of synchronization with auditory or visual cues. Furthermore, interpersonal synchronization may have the added benefit of exposing the “student” to the natural fractal fluctuations of their “instructor's” gait.

In 2014, Marmelat et al. observed that followers were able to effectively synchronize their gait to that of leaders [21]. An interesting observation was that the fractal indexes of stride fluctuations did not differ between followers and leaders and were highly correlated. This suggests that followers match their stride time structure to that of leaders. Based on this consideration, this research group developed the concept of *complexity matching* applied to interpersonal gait synchronization [22]. First introduced by West et al. [23], complexity matching means that the information exchange between complex networks is maximized when their complexities are similar. In other words, when a complex network stimulates a second network, the response depends on the matching of their complexity. This phenomenon is also known as 1/f resonance. Using innovative statistical methods, Almurad et al. [22] provided convincing evidence that complexity matching is a plausible explanation for how individuals attune their gait to each other. They also showed that arm-in-arm walking induces a stronger gait attunement than side-by-side walking without tactile contact between partners. The importance of tactile stimuli to enhance gait synchronization has also been highlighted in other studies [24, 25].

In a study published in 2018, the same research group further tested complexity matching in older individuals [26]. They hypothesized that when an older person walked in close synchrony with a younger partner, the complexity matching effect would improve the older person's gait complexity. They tested this hypothesis in two groups of 12 people. One group walked in pairs side-by-side with an experimenter, without voluntary synchronization. The second group walked arm-in-arm with voluntary synchronization. The gait training consisted of 44 walking sessions for a total of about 67 km over four weeks. The results showed that, in the arm-in-arm group, gait fluctuations were dominated by complexity matching. They observed a restoration of complexity in the older participants after three weeks, and this effect persisted two weeks after the end of the training session. These results were further confirmed in another study with different participants [27]. Although these results are promising, the restoration of a healthy complex gait does not necessarily imply a relevant improvement in the motor function. Further studies are needed to examine the effects of arm-in-arm gait training on clinically relevant measures of walking ability.

### Rationale and objectives

A high level of physical activity is the cornerstone of healthy aging. Falling is a major problem for older adults and appropriate exercise can help prevent falls. However, the type of exercise—and the optimal way to deliver it—that maximizes efficacy is still unknown. The most efficient exercise intervention should have the following characteristics: it should have a specific effect on reducing fall risk, it should be of sufficient intensity to improve cardiovascular fitness and muscle strength, it should be safe, it should not incur high costs, and it should be sufficiently enjoyable and motivating to induce a high adherence and compliance. The present project aims to test an innovative exercise intervention that may have all these advantages.

The general idea of this research project is to confirm the results of Almurad et al. [26, 27] in a larger sample. The main modification to Almurad's protocol is that the “gait teacher” during arm-in-arm walking will be a younger person (different for each older participant) rather than a member of the study staff (the same for each older participant). We hypothesize that having a family member or younger person participate in the training program may have positive effects on social interactions and exercise adherence. The intervention will consist in four weeks of 30 min arm-in-arm gait training three times per week. We will focus on clinically relevant outcomes to highlight potential benefits of the intervention in terms of gait quality, balance, fall risk reduction, physical activity levels, mood, and well-being. We will evaluate the superiority of arm-in-arm gait training compared to standard walking (without gait synchronization) using a randomized-controlled design. The perception of the intervention by older participants will be explored through a qualitative analysis.

### Trial design

The AAGaTT study, is a monocentric, two-arm, open-label, randomized controlled trial. The superiority of arm-in-arm synchronized gait training over standard walking (i.e., walking side-by-side without gait synchronization) will be assessed after four weeks of 30-min training 3 times per week.

## Methods/design

### Study setting

The study will take place at the Haute-Ecole Arc Neuchâtel, which is a university of applied sciences located in Western Switzerland (French speaking part). Study participants will be recruited from the urban and peri-urban area of Neuchâtel, a small town of 46,000 inhabitants. In 2021, the proportion of older people (> 65 years) represented 19.6% of the population [28]. Switzerland is a country with a high human development index (HDI [29]); Switzerland’s HDI value for 2019 is 0.955, placing it in second place out of 189 countries and territories [30], reflecting its high standard of living and high life expectancy.

### Eligibility criteria

Eligible older participants should be > 70 years of age and able to walk continuously for 15 min without assistance. In addition, they must have experienced a fall in the year prior to recruitment. The decision to set the lower age limit at 70 years is strategically aimed at a population group that is particularly prone to falls. It is well documented that the incidence of falls increases significantly after the age of 70 [31], a trend that is particularly pronounced among older men [32]. In addition, the use of 70 as the cut-off age for older adults is consistent with recent large-scale population studies of physical activity and fall risk [33, 34]. The participants must not have severe gait disorders of orthopedic or neurological origin (such as lower limb amputation or severe hemiparesis). Mild gait abnormalities (e.g., mild limp due to knee osteoarthritis or mild gait asymmetry due to limited hemiparesis) will be tolerated.

Eligible younger participants must be older than 18 years old and younger than 40 years old. The younger participant must exhibit no severe gait disorders from musculoskeletal or neurologic origin.

The exclusion criteria are as follows: inability to follow the procedures of the study, e.g., due to language problems, psychological disorders, dementia, etc.; inability or contraindications to undergo the intervention under study. We will not recruit vulnerable people.

### Interventions

For both groups (intervention and control), a typical gait training session will consist in 30 min of side-by-side walking. The duration of the session may be shortened in case of fatigue or pain. The exact duration of the session will be timed. The session will take place on an indoor track to avoid adverse weather conditions. For the intervention group, the participants will be asked to walk arm-in-arm while synchronizing their steps [26]. They will have to agree on a comfortable pace for the older participant. Similarly, for the control group, the participants will walk side by side without contact and without any instructions to synchronize their gait.

The gait training session will be repeated three times per week for four weeks. The last session of each week will be completed with an evaluation session to assess gait quality (see Tables 1 and 2).

**Table 1:**
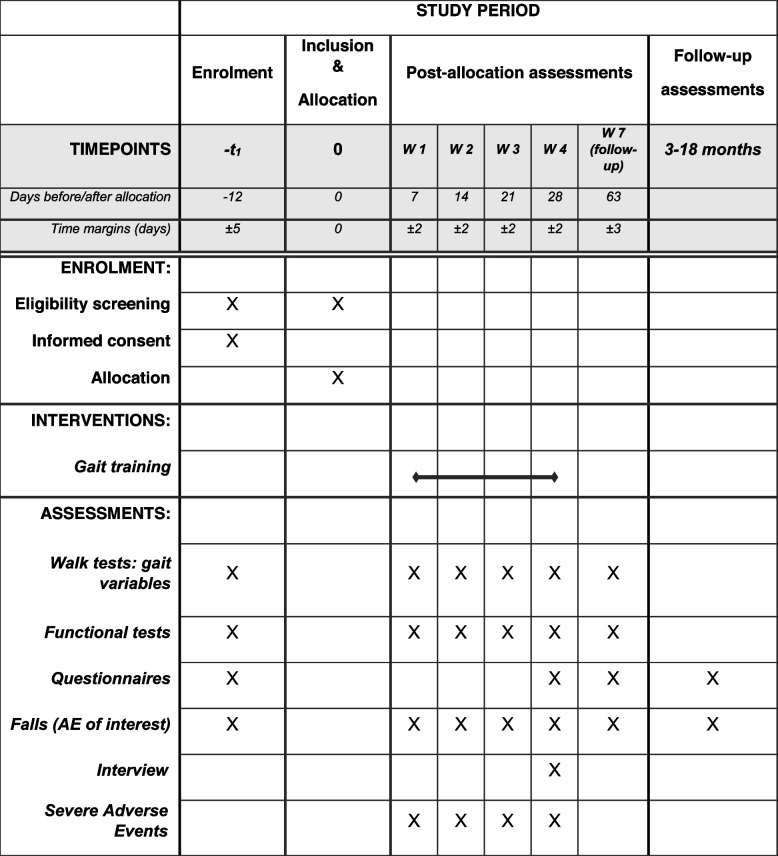
Schedule of enrolment, interventions, and assessments (SPIRIT guideline)

**Table 2:** Description of data collection tools

Functional tests	*Timed Up and Go (TUG) test*	The Timed Up & Go test measures, in seconds, the time it takes a person to stand up from a standard armchair, walk a distance of 3 m, turn around, walk back to the chair, and sit down again [35]. At the beginning of the test, the subject is seated in an armchair. The instructions are: "On the word GO, you will stand up, walk to the line on the floor, turn, walk back to the chair, and sit down. Walk at your normal pace.” The subject wears his or her normal shoes and may not be assisted by another person. There is no time limit. They may stop and rest (but not sit down) if they need to
	*Unipedal stance test / single limb stand time*	The Unipedal stance test [36] is a measure of the ability to stand on one leg and maintain balance. Participant instructions are: “I am going to time how long you can stand on one leg for each leg, keeping your hands on your hips. We will randomly pick one leg to start. I will start the clock when your foot lifts off the floor. You may balance using any method that you like as long as you are on one leg and the other leg is unsupported. I will stop the clock either when your foot touches the ground, your hands come off your hip, you move your standing foot or the opposite foot braces against the standing leg.” The test will be performed with the participant’s shoes off. The test is repeat three times for each leg. The result is the average of the scores
Gait test		The participants will walk twice (back and forth) along a corridor measuring about 200 m (no stairs and no curve, total 400 m). They will walk at their preferred (most comfortable) speed. Two small (42.2 × 31.6 × 15 mm, 15 g), high-accuracy (± 8 g, 16 bit, 256 Hz), wearable inertial sensors (Physilog 6S, Gaitup, Switzerland) will record accelerations at foot and lumbar level. The sensors are synchronized together via Bluetooth. The Acceleration signals will be used to compute several parameters characterizing the gait quality of the participants (see below for more details). We expect to record about 300 steps per session
Questionnaires	*Physical activity level over the last week*	QAPPA (4 questions) [37]. The questionnaire distinguishes between low, moderate, and vigorous physical activity. The time spent per week (in minutes) is multiplied by 8 for vigorous activities and by 4 for the moderate activities to give the level of metabolic equivalent of task (MET-min/week). A low level of physical activity corresponds to the cases that do not meet the classification for moderate or vigorous activity
	*Mood*	GDS (15 questions) [38]. The Geriatric Depression Scale (GDS) is a self-rating scale designed for rating depression in older adults. The GDS questionnaire is composed of 15 questions. 10 of the questions indicate the presence of depression when answered positively, the 5 others indicate the presence of depression if answered negatively
	*Quality of life and wellbeing*	ICECAP-O (5 questions) [39]. The Investigating Choice Experiments for the Preferences of Older People (ICEPOP) CAPability (ICECAP-O) is a capability-based measure of the general quality of life of older people (≥ 65 years old). The ICECAP-O measures 5 attributes: Attachment (love and friendship), Security (future), Role (doing things that make you feel valued), enjoyment (enjoyment and pleasure), and control (independence). Each attribute has 4 level of answers. The ICEPCAP-O range on a scale from 0 to 1
	*Fear of falling*	FES-I 16 questions [40]. The falls efficacy scale international (FES-I) questionnaire measure the “concern about falling”. The questionnaire is composed of 16 questions with 4 choices valued from 1 to 4. The minimum score is 16 and the highest 64

### Outcomes

The primary endpoint will be the difference in walking speed between the intervention and the control groups at the end of the intervention (week 4).

Secondary endpoints will be pre-post differences (week 4 versus baseline): we will compare the efficacy of training on walking speed, functional tests, gait parameters, and other psychological and heath related variables with results from the literature [8, 41–47]. We will also examine the differences between the groups at follow-up (week 7). Carry-over effects (week 4 vs. week 7) will also be assessed. Finally, we will compare the incidence of falls in both groups over 18 months after the intervention.

The primary outcome will be the walking speed. The speed at which older people walk is a reliable measure of functional capacity with a well-documented predictive value for important health-related outcomes [48]. Walking speed has been shown to be predictive of many outcomes including: functional dependence and need for personal care [49], falls [50–52], cognitive decline [53], cardiovascular-related events [54], and all-cause mortality [55]. Measurement of walking speed has been shown to be valid [56], reliable [56, 57], and sensitive [58, 59]. It is also well established that slow walking is associated with poorer gait quality [60] and an increased risk of falls [50]. Because of its predictive power and its interesting clinimetric properties, walking speed has been called the “sixth vital sign” [61]. Walking speed is therefore often used as an outcome measure for rehabilitation efficacy, for example in stroke patients [62, 63], in the older people [64], or in patients with Parkinson’s disease [65, 66].

The secondary outcomes will be:

o **Gait variables.** Average step frequency and step length. Step symmetry and stride regularity. Gait stability (local dynamic stability). Fractal index. (See below for further details).
o **Functional tests.** Timed up-and-go [35]. Unipedal stance test [36, 67].
o **Other variables.** Depression (15-item geriatric depression scale, GDS [38]). Fear of falling (Falls efficacy scale international, FES-I [40]). Perceived physical activity level (Questionnaire d’activité physique pour les personnes âgées, QAPPA [37]). Quality of life; Investigating Choice Experiments for the Preferences of Older People (ICEPOP) programme, capability measure of general quality of life: the ICEPOP CAPability (ICECAP-O) instrument [39]. Adherence (number of participants withdrawing from the study). Retrospective fall rate (falls occurring over one year before the study). Prospective fall rate (falls occurring over 18 months after the study).
o **Qualitative results.** Narrative feedback after transcription of semi-structured interviews. Quotes will be chosen to illustrate themes that were common, or that were a summary of a topic.

### Sample size

We will recruit 66 dyads (i.e., 66 older people and 66 younger relatives acting as “gait instructors”). The determination of the sample size is based on 1) the estimated ability of the research team to conduct the experiment over the planned three-year period, and 2) the availability of the indoor walking circuits, and 3) the estimated power of the study. For the main outcome (walking speed), a standardized effect size of 0.5 is considered as substantial and meaningful [68]. The minimum sample size according to [68] is 37 to 42 participants. Therefore, the study sample size (66) is sufficient to detect a potential intervention effect with a comfortable margin.

In addition, we performed a sensitivity analysis using G*power [69]. A sample size of 60 dyads was used to account for 10% loss to follow-up. We tested the ability of multiple linear regression with walking speed as the response variable to detect a partial increase in R^2^. Three parameters were considered (group, speed at baseline, and total duration of training). We sought to predict the sensitivity of the model to the group effect. With 80% power and a 5% significance level, the predicted minimum detectable effect size (Cohen's f2) is 0.14, a small effect size on Cohen's scale.

In analogy to the effects of auditory cues on gait speed and step length (meta-analysis in older adults, standardized effect size Hedges'g ~ 0.6 [8]), 26 measurements should be sufficient to detect a positive pre-post effect. In fact, 26 paired observations are required to achieve 90% power and a 5% (one-sided) significance level to detect an effect size of 0.6 (paired t-test). Therefore, the target sample size of 33 participants in the intervention group seems appropriate to detect pre-post effects of arm-in-arm gait training, assuming a similar effect as walking following cues.

### Recruitment

Participants will be recruited through several publicity campaigns aimed at the general population. The dyads will also be recruited through a campaign aimed at HE-Arc and university students, and associations of older people, and through contacts with medical institutions in the Neuchâtel area. In order to facilitate the recruitment and to allow access to the general documents related to the recruitment at any time, we will use the institutional website as a showcase page where people will be able to find the recruitment announcement and all the documents used in the recruitment process. Interested parties will also be able to contact the research staff directly through this page. As an alternative method of recruitment, information sessions will be held to explain the study to several candidates at the same time. After a quick check of the suitability of the candidate's profile, the informed consent form will be sent by email or, if necessary, by mail.

The candidate will be contacted (by phone) a few days later to provide further explanations, answer questions, and—if the candidate is still interested in participating—to schedule an initial meeting at which participants will sign the informed consent and then take baseline measurements (for older participants).

### Assignment of intervention and blinding

After the baseline assessment, participants will be randomized to either the control or the intervention group in a 1:1 ratio according to a computer-generated list. A researcher will generate the allocation sequence using the R package *blockrand* [70] independently of study staff. Group allocation will be performed using a block randomization procedure with randomly permuted block sizes (4, 6, 8). Allocation assignment will be concealed from the enrolling investigators using the electronic data capture software REDCap [71, 72]. After the randomization list is uploaded by an independent person not involved in the study, REDCap will mask the allocation sequence to all study staff. A special REDCap tool will reveal the group assigned to the current participant when all baseline data have been collected.

Due to the nature of the intervention, participants and the study team will not be blinded to group membership (open trial). To mitigate this bias, we will use objective assessment methods such as gait quality assessment with accelerometers and validated questionnaires. In addition, both interventions (arm-in-arm and normal walking) will be presented to participants as equally effective in improving fitness.

### Experimental procedures

At the time of enrollment, we will ask older candidates to authorize contact with their referring physician to obtain a health certificate attesting that there is no contraindication to walking. Note that the study does not require access to the participant's medical record.

Older participants will come to the HE-Arc campus for baseline measurements. They will perform functional and gait tests, and complete questionnaires (see Tables 1 and 2). The baseline session will last about one hour.

Three times a week for four weeks, the participant and his or her "gait instructor" (a younger person less than 40 years old) will train for 30 min. One break will be allowed. Sessions may be shortened due to participant fatigue.

The gait and functional tests will be repeated before the third session of each training week (week 1 to week 4) and during the follow-up (week 7). In addition, both participants will wear accelerometers during the third training session to assess the degree of gait synchronization.

Participants will be invited to complete the questionnaires during the last session of week 4 and during the follow-up at week 7 (see Table 1).

A randomized sample of participants will be invited to participate in a semi-structured interview after the last training session of week 4. The interviewer will be a research assistant. The interview duration will be 20–30 min. The interviews will be audio taped and verbatim transcriptions will be realized.

**Fig. 1:**
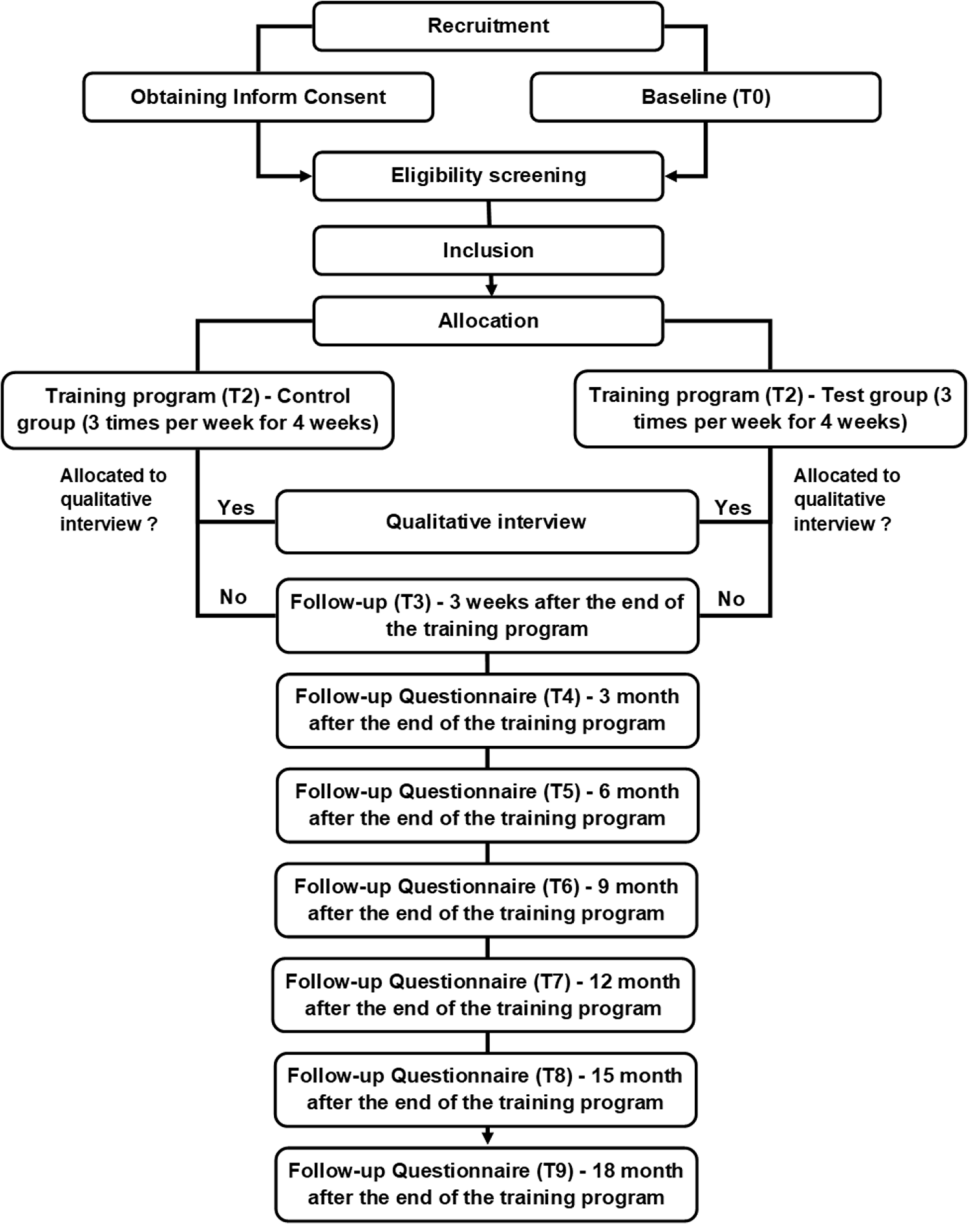
Flowchart of the participants’ timeline

The number of falls in the previous year will be recorded at baseline. Every 3 months after the end of the training, participants will receive an email inviting them to complete the QAPPA questionnaire (see description of measurement tools) and to indicate whether they have fallen in the last 3 months. The detailed participant timeline is shown in Fig. 1 and Table 1.

### Data collection, quality measures, and confidentiality

An electronic data capture system (REDCap) will be used to manage the case report form (CRF) of the study (eCRF). Study documentation (e.g., standard operating procedures, correspondence, etc.) will be organized according to the recommendations of the good clinical practice (trial master file) [73]. The principal investigator will train the study staff in all important aspects of the project, with emphasis on data management.

Data will be collected by the research team (principal investigator and research assistant) at six different time points throughout the study: enrollment, weeks #1, #2, #3, #4, and week #7 (follow-up). An email with a link to two follow-up surveys (retrospective falls and physical activity during the last seven days) will be sent to all participants every three months until 18 months after the end of the training program (week 4). In case of non-response, the research assistant will call each participant individually to remind them to complete the surveys. Follow-up will be discontinued if two falls occur within 12 months, as the 12–18-month data will only be used for survival analysis (time to first and second fall).

Given the low-risk nature of the study and to minimize the collection of medical data from participants, we will only monitor whether a fall or walking-related conditions (muscle soreness, joint pain, foot pain) occur during the gait training (week #1-#4), either directly during the training sessions or under other circumstances. Therefore, all walking-related adverse events (AEs) or serious adverse events (SAEs) occurring during the gait training will be reported in a special REDCap questionnaire.

A participant may be withdrawn from the study for the following reasons: withdrawal of informed consent, conditions that prevent continuation of gait training and completion of other study tasks (illness, accident, death), and loss of contact (participant does not respond to e-mail or telephone). If possible, we will perform a final measurement session at the time of withdrawal to assess the current status of the participant. In the event that an accompanying participant withdraws from the study, whether voluntarily or due to external circumstances, a replacement volunteer will be recruited to ensure continuity of the gait training program.

The quality of the study data will be reviewed by an external monitor. The prospective institution is the "Unité de Conseil et de Coordination de la Recherche Clinique UCCR-CRC / CHUV-UNIL, Lausanne VD". The monitoring plan includes review of mandatory documents, initiation and follow-up visits, and monitoring of the eCRF. Source data and documents as well as the eCRF will be accessible to the monitors and questions will be answered during the monitoring visits.

In the case of substantial changes in the study design and organization, the protocol and relevant study documents will be submitted to the ethics committee for approval prior to implementation. Deviations from the protocol to protect the rights, safety, and welfare of participants may be made by the principal investigator without prior approval of the ethics committee. Such deviations will be documented and reported to the ethics committee as soon as possible. Clinicaltrial.gov data will be updated accordingly.

Study and participant data will be held in the strictest confidence and will be available only to authorized personnel who need the information to perform their duties in connection with the study. A confidential, password-protected list of participants and their contact information will be maintained. On study-specific documents, participants will be identified only by a unique participant number. At the end of the study, the final dataset will be anonymized. A complete de-identification process will be performed using REDCap's anonymization capabilities.

### Data analysis and statistics

The gait patterns of the older participants will be evaluated at baseline, at the end of each training week (i.e., 4 times), and at week #7. The average walking speed will be computed from walking time (using a stopwatch) and distance.

Heel strikes (and thus stride intervals) will be measured from the foot acceleration signal. The reliability of detecting gait events from a foot-mounted accelerometer has been shown to be excellent in a recent meta-analysis [74]. The number of steps, average step frequency and length, and walk ratio [75], will be computed. Detrended fluctuation analysis (DFA) will be used to compute the fractal index of the time series of stride intervals.

From the trunk acceleration signal, step symmetry and stride regularity will be assessed using the autocorrelation function (ACF) method [76, 77]. Movement intensity will be assessed using root mean square (RMS) acceleration variability. We will also calculate the RMS ratio, which is an index of dynamic equilibrium [78]. A multidimensional attractor will then be constructed using the time delay embedding method. LDS (short-term DE) and ACI (long-term DE [79]) will be computed using the average logarithmic rate of divergence between neighboring trajectories (Rosenstein’s algorithm [79–82]). For further details on the validity, justification, and calculation of the gait quality variables, please refer to this previous study, section D [83]. This study also includes a reliability analysis of the different gait parameters (ACF, RMS, LDS) assessed under free-living conditions.

Gait synchronization within the walking dyad will be assessed with windowed detrended cross-correlation analysis (WDCC [84]) using the time series of stride intervals measured by the foot accelerometers during the last training sessions of each week.

Regarding the qualitative study, a qualitative grounded theory approach will be used, in which the participants are seen as experts on their own experiences. The general interview guide approach will be used [85]. First, a preliminary interview guide will be developed. The open-ended questions will concern attitudes and personal experiences with gait training. We will seek information about motivation, enjoyment, and ease. The preliminary guide will be tested and iteratively improved using the first four interviews. The final guide will then be applied to the next group of participants. We expect to interview 15 older participants (from both groups), depending on theme saturation.

R and Matlab will be used for statistical analyses. Medians and interquartile ranges of the collected variables will be calculated as descriptive statistics. Boxplots will be used to highlight the distribution of variables. In terms of inferential statistics, the main endpoint will be assessed using multiple regression analysis. The dependent variable will be walking speed at the end of the intervention (week #4) and at week #7. Group membership (independent variable) will be coded using a dummy variable (0/1 for intervention or control group). Models will be adjusted (covariates) for baseline walking speed and total walking time across all training sessions. The choice of covariates will be guided by their potential association with the main outcome (final walking speed).

As secondary analyses, we will also apply regression models for the other outcomes (gait parameters, functional tests, questionnaire scores). Paired t-tests and effect sizes (Hedges’s g) of primary and secondary outcomes will be computed to evaluate pre/post and carry-over effects. Linear mixed models will be also fitted to assess how the differences between groups evolve across the four weeks of training.

The method for analyzing the follow-up results (time to first and second falls) will include both standard analyses and survival analyses. For the standard analysis, after one year of observation, we will separate participants into those who fell and those who did not. Logistic regression will be performed with fall status as the dependent categorical (two-level) variable and group (control/intervention) as the independent variable. As exploratory analyses, we will also test whether other parameters (gait quality, questionnaire score) influence the probability of falling. Regarding survival analyses, we will use the same analysis as presented in a study on fall risk in patients with Parkinson's disease [86]. By performing survival analyses (Kaplan–Meier tests), we will estimate whether group (control / intervention) is associated with time to first and time to second fall. In addition, Cox regression analyses will assess the association between increased fall risk (hazard ratio for experiencing a fall in any month) and gait parameters.

The significance level will be set at 0.05. Any deviation from the original statistical plan will be described and justified in the final study report.

## Discussion

Overall, the risk–benefit balance of the AAGaTT trial is very favorable.

In terms of individual risks, because they will be walking with a younger partner, older study participants will be exposed to a fall risk that is lower than normal walking. In addition, we will exclude individuals with a contraindication to moderate physical activity or walking. Regarding privacy, we will apply strict data management rules to reduce the risk of unauthorized data access or unwanted identification of participants. In addition, we will not collect any data about participants' health status other than their walking ability and balance. As a result, participants are exposed to minimal risk.

In terms of individual advantages, increasing physical activity has strong benefits. The addition of 90 min of walking per week (60% of the WHO recommendation) will improve strength and cardiovascular fitness. Therefore, participants in both groups will benefit from participating in the study.

In terms of societal benefits, the number of people in Switzerland over the age of 80 will increase from 460,000 in 2015 to 840,000 in 2035 (+ 83%). This will inevitably place a significant burden on society and the health care system, which will be difficult to bear if the efficiency of care for the older persons is not improved. Increasing the physical activity of the older population is a preferred approach to promoting healthy aging [87]. In addition, the increasing incidence of falls among seniors is worrisome. In the US, the absolute number of deaths due to falls almost tripled in the last decade, from 8,613 in 2000 to 25,189 in 2016 [88]. Therefore, there is an urgent need to develop innovative and cost-effective approaches to reduce fall risk and increase physical activity in the older population.

Although adapted exercise can specifically reduce the risk of falls, lack of motivation, and other psychological and social factors (depression, social isolation, and cognitive ability) are barriers that prevent many older people from reaping the benefits of exercise. We believe that involving younger people may improve exercise adherence. Indeed, exercising with a partner may provide reassurance, support, and motivation. The results of the qualitative analysis will allow us to consider the participants' feelings about the gait training program.

In conclusion, the expected benefits of arm-in-arm gait training for older people are multiple: reduction of fall risk, improved cardiovascular fitness, increase in muscle strength, safe treatment with no adverse effects, high adherence due to increased motivation, and low-cost intervention. The AAGaTT results will provide further insight into the efficacy and effectiveness of this novel training method.

## Data Availability

Data sharing is not applicable as this article describes a study protocol. At the end of the study, according to the institutional policy about data sharing (Swissuniversities open research policy [90]), we will make final datasets openly available on appropriate digital data repositories at the latest at the time of publication of the results. We will restrict data sharing only to ensure novelty of publication. Except for this, we will share data as widely as possible using Creative Commons licenses. The present protocol and other future articles follow the principles of SPIRIT [91] and CONSORT [92] guidelines. Co-authorship on any of the publications will be based on contribution to the study and manuscript according to the criteria of the International Committee of Medical Journal Editors. No professional writer will be hired.

## Abbreviations

AAGaTT: Arm-in-arm gait training trial
ACF: Autocorrelation function
AE: Adverse event
CHUV: Centre hospitalier universitaire vaudois
CTU: Clinical trial unit
DFA: Detrended fluctuation analysis
eCRF: Electronic case report form
FES-I: Falls efficacy scale international
GDS: Geriatric depression scale
HDI: Human development index
HE-Arc: Haute Ecole Arc
ICEPOP-O: Investigating choice experiments for the preferences of older people (ICEPOP) CAPability (ICECAP-O)
LDS: Local dynamic stability
MET: Metabolic equivalent of task
QAPPA: Questionnaire d'activité physique pour les personnes âgées
REDCap: Research electronic data capture
RMS: Root-mean-square
SAE: Serious adverse event
TUG: Timed up-and-go
WDCC: Windowed detrended cross-correlation
WHO: World health organization
